# Autonomic modulation and skeletal muscle oxygenation with intermittent low-load blood flow restriction knee extension

**DOI:** 10.3389/fspor.2025.1515412

**Published:** 2025-03-13

**Authors:** Andrew R. Garner, Jacob D. Fanno, Ryan McGrath, Jacob Erickson, Kyle J. Hackney

**Affiliations:** ^1^ Department of Health, Nutrition, and Exercise Sciences, North Dakota State University, Fargo, ND, United States; ^2^ Department of Human Performance, Sport, and Health, Bemidji State University, Bemidji, MN, United States

**Keywords:** blood flow restriction, autonomic modulation, heart rate variability, skeletal muscle oxygenation, resistance exercise

## Abstract

**Introduction:**

This investigation determined if an acute bout of low-load knee extension (KE) with intermittent blood flow restriction (BFR) influenced autonomic modulation and skeletal muscle oxygenation (SmO_2_%).

**Methods:**

Fourteen physically active males completed three different sessions: one-repetition maximum (1RM), KE with BFR (BFR-KE) at 20% 1RM (cuff pressure=143 ± 13 mmHg), and KE with free blood flow at 20% 1RM (Control-KE). Heart rate variability (HRV) metrics: logarithmically transformed (ln) square root of the mean differences of successive R-R intervals (lnRMSSD), high frequency power (lnHF), and low frequency power (lnLF), as well as SmO_2_%, and rating of perceived exertion (RPE) were measured. Repeated measures analyses of variance were used to analyze HRV metrics and SmO_2_%, while a paired *t*-test was used to analyze RPE. A significance level of *P* *<* 0.05 was used for analyses.

**Results:**

From baseline to 15 min post-exercise lnRMSSD decreased in both BFR-KE and Control-KE (4.34 ± 0.43–3.75 ± 0.82 ms, *P* = 0.027). Thereafter, lnRMSSD (+7%), lnHF (+8%), and lnLF (+7%) increased from 15 to 30 min post-exercise in both BFR-KE and Control-KE (*P* < 0.05). BFR-KE reduced SmO_2_% in the vastus lateralis compared to Control-KE (36% vs. 53%; *P* < 0.001). RPE was greater in BFR-KE (7.0 AU) compared to Control-KE (4.5 AU; *P* *<* 0.001).

**Conclusion:**

Unilateral BFR exercise with individualized cuff pressure and intermittent application facilitated greater localized muscular stress and perceptual effort, but there was no influence of vascular occlusion on post-exercise autonomic modulation compared to volume-matched exercise with free blood flow.

## 1 Introduction

Based on a recent review it is suggested that resistance exercise acutely modulates autonomic nervous system control during the post-exercise recovery period by attenuating vagal tone (e.g., parasympathetic activity) (1). These acute alterations can be measured using time domain and frequency indexes of heart rate variability (HRV) (2). The time domain metric, square root of the mean differences of successive R-R intervals (RMSSD), is used as a measure of vagal activity (3). Regarding the frequency domain, high frequency power (HF) is used as a measure of vagal activity, while low frequency (LP) power is a measure of both vagal and sympathetic activity (4). The calculated LF to HF ratio indicates sensitivity for sympathovagal dominance (5). With high-load resistance exercise acute vagal withdrawal (↓HF) has been reported for 25–35 min post-exercise (6, 7) with additional suppression at 24 h (8). Several factors are related to these alterations which include catecholamine release, metabolite accumulation, plasma volume shifts, and activation of the arterial baroreflex (1).

An alternative to high-load resistance exercise is low-load blood flow restriction (BFR) exercise (9). BFR exercise uses partial vascular occlusion by a pneumatic cuff or tourniquet (10). The cuff is placed at the most proximal portion of the active limb and lower exercise loads [20%–40% of one-repetition maximum (1RM)] are utilized (11). BFR exercise facilitates acute homeostatic disturbances, greater metabolic activity, and increased molecular signaling to enhance exercise adaptations (12). Reduced skeletal muscle oxygenation in muscle groups distal to the cuff placement has been consistently shown (13, 14). As BFR exercise is becoming more common in clinical settings (15–17), it is critical to examine the influence vascular occlusion on post-exercise HRV given a decrease in vagal modulation is associated with higher all-cause mortality (18), cardiac events (19), and ventricular arrhythmias (20).

Few investigations have evaluated changes in HRV metrics following BFR exercise (21, 22). Comparisons are generally limited to high-load resistance exercise vs. low-load BFR and studies have only evaluated continuous BFR exercise methodology (21, 22). Continuous BFR exercise refers to when the cuff remains inflated during the rest interval following a set as opposed to intermittent BFR where the cuff is deflated during the rest interval following a set (11, 23). There are also differences in the application of cuff pressures and exercise load across studies loads utilized. For example, Okuno et al. (2014) reported high-load (80% 1RM) leg press exercise induced greater vagal disturbance compared to continuous BFR leg press exercise (40% 1RM, standard pressure 100 mmHg, cuff width=14 cm, length=70 cm) (21). Tai et al. (2019) showed both high-load (70% 1RM) and low-load BFR (30% 1RM) bench press exercise (using perceptual rating for cuff pressure of 7 of 10 using 77 mm-wide wraps), decreased vagal activity 30 min post-exercise, with greater disturbances in high-load resistance exercise (22). Given few reports to how BFR exercise may influence HRV metrics and application of BFR methodology is variable, the purpose of this study was to determine if an acute bout of low-load intermittent BFR exercise affects autonomic modulation during a unilateral knee extension (KE) exercise at the same relative intensity and volume. The decision on “continuous” or “intermittent” application may be largely up to the clinician or research scientist, however, the tolerability of the exercise is also a significant factor for patients and/or athletes. Our laboratory has found acute upregulation of circulating progenitor cells from a single acute session (14) and chronic strength gains over time (24) with intermittent BFR application. Others have also shown no differences in whole blood lactate responses, plasma volumes, or muscle activity following intermittent vs. continuous BFR applications (25). However, hemodynamic responses and perceived exertion measures are generally lower with intermittent BFR application (26). Thus, intermittent BFR application may be more tolerable for patients or athletes using the methodology. We hypothesized BFR exercise, even with intermittent application, would lead to greater perceptual effort, increased localized muscle hypoxia, and larger disturbances in post-exercise autonomic modulation compared to knee extension with free blood flow.

## 2 Methods and measurements

### 2.1 Participants

A within-participant, crossover design was utilized for this investigation. Adults aged 18–35 years were eligible if they had at least 6 months of strength training experience and were at low risk of blood clotting. Exclusion criteria were any known cardiovascular, pulmonary, metabolic diseases, previous diagnosis of exertional rhabdomyolysis, sickle cell anemia, deep venous thrombosis, recent surgery, body mass index of >35 kg/m^2^, or significant orthopedic pain. Participants who self-reported as current smokers, taking heart medications, or had implanted medical devices were excluded.

The Physical Activity Readiness Questionnaire for Everyone (PAR-Q+) (27), a deep venous thrombosis assessment (28), and a study specific questionnaire were used for screening. Adequate sample size was estimated from analysis of variance with repeated measures *a priori* using partial eta squared effect size (0.474) for an interaction effect for LnRMSSD (22), resulting in a predicted sample (effect size f = 0.94, alpha 0.05, power = 0.80) size of *n* = 10. As such, our final analytical sample included 14 participants which was representative of previous studies conducted on HRV and exercise (12, 21, 22, 29). Participants completed three exercise sessions in the following order: 1RM, KE with BFR at 20% 1RM (BFR-KE) and KE with free blood flow at 20% 1RM (Control-KE). Each session was separated by at least 72 h. Before each session, participants maintained the same dietary pattern and avoided caffeine and alcohol for up to 12 h before exercise testing. Participants avoided high-intensity exercise for at least 48 h before each session. Written informed consent was provided by participants before entering study and the university's institutional review board approved all protocols.

## 3 Methods and measures

### 3.1 Descriptive characteristics and one repetition maximum (1RM)

Age was self-reported. A stadiometer (Seca 213, Chino, CA) and floor-level scale (Detecto, Webb City, MO) measured height (cm) and body mass (kg), respectively. Body mass index was calculated as kg/m^2^. The limb circumference (cm) of the participant's right thigh was measured with a Gulik tape measure at the maximum portion of the upper thigh, between the greater trochanter and lateral condyle of the femur, in order to select the appropriate cuff size for each individual. The measurement was performed in a standing position with weight partially shifted to the left limb and the right knee slightly flexed. Prior to 1RM assessment, participants completed a 5-minute warmup on a cycle ergometer. A KE exercise machine (Cybex International, Owatonna, MN) was used to assess 1RM. A modified protocol from Okuno et al. (2014) was used to estimate 1RM on the KE exercise to facilitate efficiency (21). Participants were asked for their bilateral predicted 5-repetition maximum KE via survey. Subsequently, the equation [1RM = −0.46 + (0.79 × 5 reps) + (1.08×load kg)] was used to predict each participant's bilateral 1RM (30), which was then divided in half to estimate a unilateral 1RM for each leg. 1RM was then tested for each leg using 5 sets with 3-min rest intervals between each set. Before testing, KE machine adjustments were made for each participant based on limb anatomy and were recorded to ensure consistency for each session. This included ensuring the lower extremity shin pad was positioned slightly above the tongue of the shoe, KE equipment lever arm was adjusted to accommodate for varying leg lengths, and the participant was able to complete a full repetition through the full range of motion. The settings were recorded using the standard numbers on the KE machine and used for subsequent sessions. Each participant underwent 1RM testing on each of their lower limbs separately. The right leg was performed first then the left.

### 3.2 Blood flow restriction and control knee extension

A BFR cuff that was 11.5 cm × 86 cm × 5 mm (Delfi Medical Innovations Inc., Vancouver BC, CA) was applied to the upper thigh of each leg to restrict venous blood flow during the BFR-KE exercise (31). After an HRV recording was taken in the supine position, the participant remained supine and a BFR cuff was applied to the upper leg and inflated on one limb to measure 100% limb occlusion pressure (LOP). After approximately one-minute rest, the opposite leg was tested. Although there is a variability in LOP with different body positions, measurement in the supine position is the most reliable (32). The lowest LOP between each leg was used to identify the 80% LOP for each participant to use for the exercise sessions. Previous research has shown that there are no significant differences between legs when 100% LOP is measured in the supine position (33) and the lowest pressure was used in this study for participant safety. Once 80% LOP had been identified, participants completed a 5-min warm-up on the cycle ergometer. Participants were then fitted with a BFR cuff on one limb at their 80% LOP. A total of 75 repetitions over 5 sets has been shown to increase muscle adaptation significantly in previous studies when BFR is applied (11). A modified protocol from Patterson et al. (2019) and Okuno et al. (2014) was used to obtain a goal of 75 repetitions on the KE exercise for each participant (11, 21). The protocol consisted of 5 sets of 15 repetitions on each leg with a 1-minute rest interval between each set. Participants completed all five sets on the right leg before switching to the left. The BFR cuff was deflated during the rest interval to ensure participant safety and allow free blood flow to the previously exercised muscles. The exercise weight was 20% of each participant's 1RM for BFR-KE.

The Control-KE consisted of the identical KE exercise protocol. Participants began with a 5-min warm-up on the cycle ergometer and then began the exercise protocol without BFR. The exercise weight was 20% of each participant's 1RM for Control-KE and matched the weight of BFR-KE. The BFR-KE was completed before the Control-KE for the reason that a participant may not be able complete all the sets or repetitions when BFR was applied. This allowed researchers to control consistency of set and repetitions completed for each session and ensure exercise volume was equal between participants; as all participants completed the prescribed sets and repetitions at 20% 1RM for both BFR-KE and Control-KE.

### 3.3 Skeletal muscle oxygenation and rate of perceived exertion

During both the Control-KE and BFR-KE sessions, skeletal muscle oxygen saturation was assessed using a near-infrared spectroscopy sensor (Moxy Monitor, Fortiori Design LLC, Hutchinson, MN) placed on the vastus lateralis (VL) muscle of the right leg. The placement of the sensor on the VL muscle was directly below and not in contact with the BFR cuff in BFR-KE and this location was replicated for Control-KE. Rate of perceived exertion (RPE) was measured immediately after the final set of exercise for BFR-KE and Control-KE and with each leg exercised using 0–10 scale (34). Subjects were asked to provide a rating of their overall effort of the session after exercising each leg. A rating of 0 was considered no effort and a rating of 10 was related to a maximal effort (34). For the BFR-KE session, RPE measurement occurred with the BFR cuff deflated following the last set. For Control-KE session, RPE occurred following the last set as there was no BFR cuff used. The RPE for each leg was averaged so that a comparison could be made between BFR-KE and Control-KE.

### 3.4 Heart rate variability

A Polar heart rate (Polar Electro H10, Kempele, FIN) chest monitor was used to measure HRV indices at baseline and post-exercise (15 min and 30 min) (35). HRV indices were measured in 5-min increments during baseline and post-exercise procedures in the supine position, which is highly reliable (36). The supine position was selected in order for HRV measurement to be more comfortable for participants over the 30 min post-exercise recovery window and to avoid posture specific changes in blood pressure or metabolic activity (36). The Elite HRV application was used to collect HRV data. Files were transferred to Kubios HRV software (Kubios HRV Standard version 3.5.0, Kuopio, FIN) in order to calculate HRV indices (via time and frequency domains). For the time domain, the square root of the mean differences of successive R-R intervals (RMSSD) was determined (3). For the frequency domain, LF power was defined as bands 0.04–0.15 Hz and HF power was defined as bands 0.15–0.40 Hz (1). Normality tests determined that RMSSD, LF and HF, were not normally distributed, and as with common practice (3), these variables to be logarithmically (ln) transformed to meet the assumptions for parametric statistics and analyzed as: LnRMSSD, LnLF, LnHF.

### 3.5 Data analysis

Continuous descriptive data, 1RM, exercise session, and occlusion pressure are presented as mean ± standard deviation. A 2 × 3 ANOVA with repeated measures was used to analyze HRV metrics. A 2 × 2 ANOVA with repeated measures was used to analyze skeletal muscle oxygenation between the BFR-KE and Control-KE. When significance was detected, additional *post-hoc* analysis was completed using Sidak to control for multiple comparisons. Partial eta squared (p*η*^2^) effect size estimations are included for interpretation as p*η*^2^ 0.2–0.12 is considered a small effect, 0.13–0.25 is a medium effect, and >0.26 is a large effect (37). RPE was analyzed using a paired *t*-test between BFR-KE and Control-KE. All analyses were performed using SPSS statistical software (IBM Corporation, Armonk, New York, USA, version 28) and an alpha level of less than 0.05 was used to determine statistical significance.

## 4 Results

The descriptive characteristics of the participants are presented in Table 1. HRV metric lnRMSSD was the only time-domain variable measured during the study (Table 2). There was a significant time effect for lnRMSSD (*F* = 7.215, *P* = 0.011, p*η*^2^ = 0.357). *post-hoc* analyses determined there was a significant decline in lnRMSSD from baseline to 15 min post-exercise (*P* = 0.027) and a significant increase from 15 min post-exercise to 30 min post-exercise with both BFR-KE and Control-KE (*P* = 0.005). There were no significant group (*F* = 0.013, *P* = 0.911,p*η*^2^ = 0.001) or group×time interaction (*F* = 0.297, *P* = 0.297,p*η*^2^ = 0.0890) for lnRMSSD.

**Table 1:** Participant descriptives and KE exercise parameters.

Variable	Mean ± SD
Age (yr)	21.50 ± 2.69
Height (cm)	177.81 ± 9.08
Body mass (kg)	81.98 ± 11.30
Body mass index (kg/m^2^)	25.85 ± 2.69
Thigh circumference (cm)	59.71 ± 5.88
KE 1RM left leg (kg)	46.22 ± 14.02
KE 1RM right leg (kg)	46.22 ± 14.02
KE exercise weight-left leg (kg)	9.70 ± 3.13
KE exercise weight-right leg (kg)	9.70 ± 3.13
100% LOP (mmHg)	179.11 ± 16.65
80% LOP (mmHg)	143.29 ± 13.32

KE, knee extension; 1RM, one repetition maximum; LOP, limb occlusion pressure.

**Table 2:** Heart rate variability metrics at baseline, 15 min and 30 min post-exercise.

Variable	Baseline			15 min post-exercise			30 min post-exercise		
	BFR-KE	Control-KE	Average	BFR-KE	Control-KE	Average	BFR-KE	Control-KE	Average
lnRMSSD(ms)	4.31 ± 0.39	4.16 ± 0.46	4.34 ± 0.43	3.72 ± 0.87	3.78 ± 0.76	3.75 ± 0.82*	4.00 ± 0.86	4.05 ± 0.60	4.03 ± 0.73^#^
lnHF(ms^2^)	7.43 ± 0.80	6.95 ± 1.34	7.19 ± 1.07	6.65 ± 0.95	6.59 ± 1.35	6.62 ± 1.15	7.23 ± 0.92	7.07 ± 1.01	7.15 ± 0.97^#^
lnLF(ms^2^)	7.42 ± 0.81	7.08 ± 1.05	7.25 ± 0.93	6.80 ± 0.92	6.82 ± 0.81	6.81 ± 0.87	7.41 ± 0.77	7.15 ± 0.91	7.28 ± 0.84^#^
LF/HF (%)	1.33 ± 0.96	1.43 ± 1.15	1.38 ± 1.06	1.49 ± 0.84	1.48 ± 0.95	1.49 ± 0.90	1.43 ± 0.93	1.59 ± 1.18	1.51 ± 1.06

*N* = 14.

* Significant time effect vs. baseline, *P* < 0.05.

^#^ Significant time effect vs. 15 min-post, *P* < 0.05. Data are mean ± standard deviation.

The frequency-domains HRV variables analyzed in the study were lnHF, lnLF, and LF/HF ratio (Table 2). There was a significant time effect for lnHF (*F* = 4.165, *P* = 0.027, p*η*^2^ = 0.243)*. post-hoc* analysis determined a significant increase from 15 min post-exercise to 30 min post-exercise with both BFR-KE and Control-KE (*P* = 0.016). There was also a significant time effect for lnLF (*F* = 4.157, *P* = 0.028, p*η*^2^ = 0.243). *post-hoc* analysis determined a significant increase from 15 min post-exercise to 30 min post-exercise with both BFR-KE and Control-KE (*P* = 0.028). No significant time effects were found for LF/HF ratio (*F* = 0.137, *P* *=* 0.873, p*η*^2^ = 0.069).

**Figure 1:**
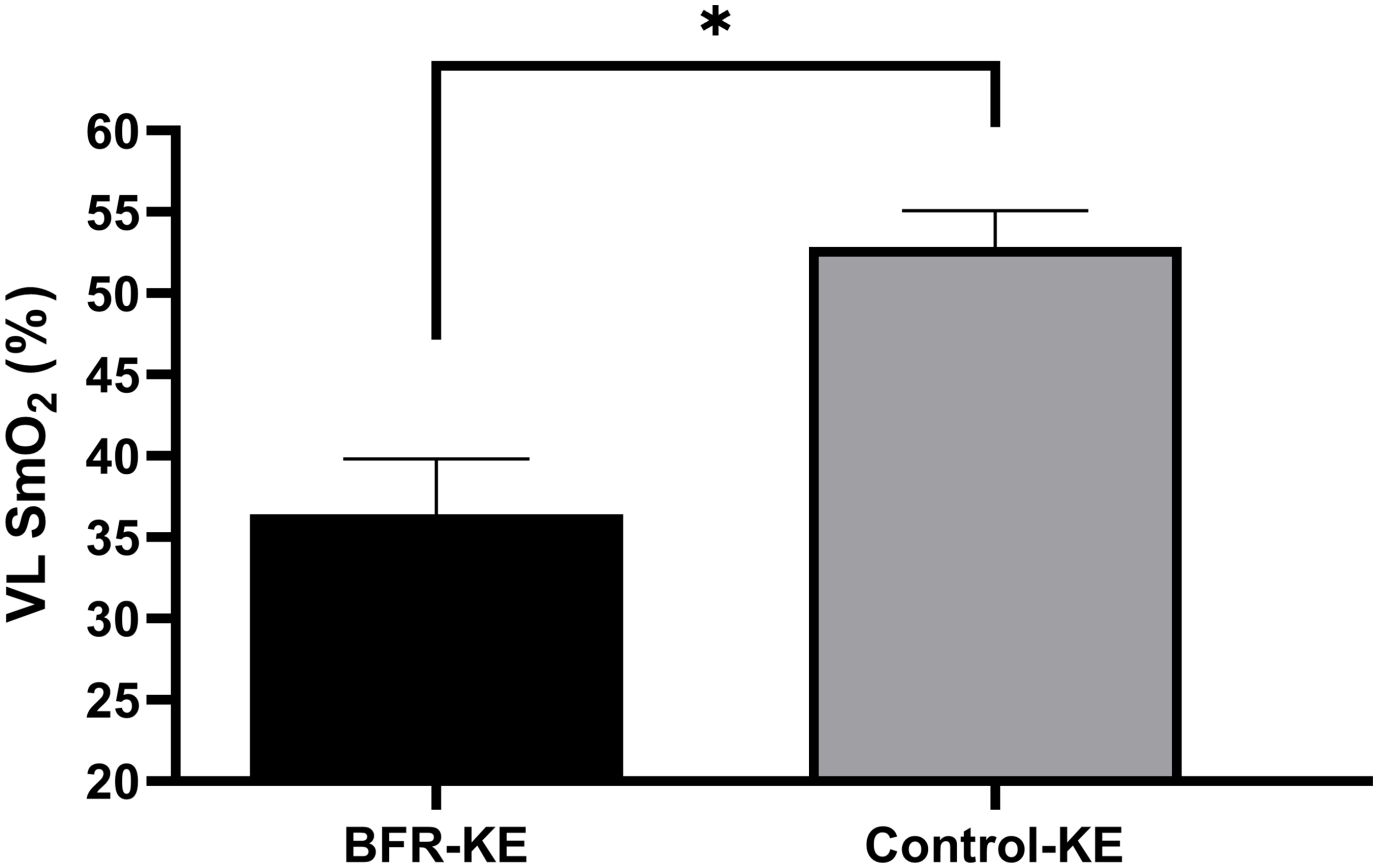
Changes in SmO_2_% with BFR-KE and control-KE. *Significant difference between groups, *P* < 0.05.

There was a significant group effect for VL SmO_2_% (*F* = 45.53, *P* < 0.001, p*η*^2^ = 0.770). *post-hoc* analysis determined VL SmO_2_% was significantly lower during BFR-KE compared to Control-KE (*P* < 0.001, Figure 1). There was also a significant group effect of oxygenated hemoglobin (*F* = 16.131, *P* = 0.001, p*η*^2^ = 0.554). *post hoc* analysis determined oxygenated hemoglobin was significantly lower in the VL muscle during BFR exercise with the rest interval compared to the control exercise with the rest interval (*P* < 0.001, Table 3). There was also a significant group effect for VL deoxygenated hemoglobin (*F* = 13.817, *P* = 0.003). *post-hoc* analysis determined VL deoxygenated hemoglobin was significantly higher in the VL during BFR exercise combined with the rest interval compared to the control exercise combined with the rest interval *P* = 0.003, Table 3). There was no significant time (*F* = 0.609, *P* *=* 0.449, p*η*^2^ = 0.045), group (*F* = 0.058, *P* *=* 0.814, p*η*^2^ = 0.004), or group by time (*F* = 1.192, *P* *=* 0.295, p*η*^2^ = 0.84) effects for total hemoglobin.

**Table 3:** Rating of perceived exertion and skeletal muscle oxygenation between BFR-KE and control-KE.

Variable	BFR-KE	Control-KE
RPE (AU 0-10)	7.10 ± 0.46*	4.50 ± 0.45
Oxygenated Hb	4.66 ± 0.42*	6.69 ± 0.26
Deoxygenated HB	8.92 ± 0.43*	6.10 ± 0.31
Total HB	12.72 ± 0.07	12.71 ± 0.08

Data are mean ± standard error.

* Significantly different between groups (*p* < 0.05).

The paired *t*-test revealed that RPE was significantly higher when BFR was applied compared to the control session [*t* (13) = 5.885, *P* *<* 0.001, Table 3].

## 5 Discussion

This investigation examined the influence of an acute session of low-load intermittent BFR exercise on autonomic modulation and SmO_2_% during a unilateral KE exercise. We hypothesized BFR would lead to greater perceptual effort, increased localized muscle hypoxia, and lead larger disturbances in post-exercise autonomic modulation. Consistent with our hypothesis, SmO_2_% declined to a greater degree with BFR-KE and this was coupled with higher RPE ratings, suggesting enhanced local muscle hypoxia and perceptual effort with vascular occlusion compared to Control-KE. Contrary to our hypothesis vagal activity decreased from baseline to 15-minutes post-exercise and increased 15–30-min post-exercise in both BFR-KE and Control-KE; with no apparent influence of vascular occlusion.

In the present study, the HRV indicator lnRMSSD significantly declined from baseline to 15 min post-exercise in both BFR-KE and Control-KE. This would suggest a disturbance in vagal activity with the low-load exercise, regardless of whether vascular occlusion was applied during the sets of muscle contractions. Thereafter, significant increases in lnRMSSD, lnHF, and lnLF were observed in both groups from 15 to 30-min post-exercise, which suggests the potential for co-activation in both sympathetic and parasympathetic nervous systems. Co-activation can occur when sympathetic activity is slow to return to baseline following a stressor, while parasympathetic activity is increased to promote recovery. This event is observed in other studies regarding “vagal rebound” in different stressful situations (38, 39). These findings suggest that exercise as a stressor can elicit a nonreciprocal response from the sympathetic and parasympathetic nervous systems (40). The underlying mechanisms of vagal rebound are unclear but some suggest exercise-related plasma volume changes and baroreflex activity may be associated with this response (41).

In comparison to the other investigations exploring continuous application of BFR and post-exercise autonomic modulation, Tai et al. (2019) examined if lnRMSSD, lnHF, and lnLF were affected following bilateral bench press using two 70 mm-wide knee wraps that were wrapped around both upper arms while using a rating scale of 7 of 10 occlusion pressure (22). Significant decreases from rest were reported in lnRMSSD for both the low-load BFR (25–30 min post-exercise) and high-load (15–20 min & 25–30 min post-exercise) exercise sessions. Tai et al. (2019) found a greater decline in lnRMSSD and lnHF during the high-load session compared to the low-load BFR session (22). In the present investigation, the p*η*^2^ effect sizes to determine group (0.043) and group×time interactions (0.024) were trivial, suggesting a large sample size (*n* = 38 to 68, respectively using G*Power 3.1.9.7) would be required to determine overall statistical significance between BFR-KE and Control-KE and statistical differences at a specific post-exercise time point (42).

Okuno et al. (2014) also investigated autonomic modulation during an acute high and low-intensity session, along with a single session of continuous low-intensity BFR at a standard pressure (100mmHg) with 40% 1RM during a unilateral leg press exercise. RMSSD decreased below resting levels for low-intensity BFR 10-min post-exercise and 30-min post-exercise for high-intensity before returning to baseline measures (21). However, post-exercise RMSSD during low-intensity exercise remained the same compared to baseline, until 24-hours. A decrease in RMSSD measures were observed during the high-intensity exercise compared to the low-intensity exercise session up to 1-hour post-exercise, while the low-intensity BFR exercise session was significantly higher compared to the high-intensity at 10 and 30-min post-exercise (21). HRV metric lnLF decreased from baseline measures to 10-min post-exercise in the high-intensity session and during the 1 and 5-h time period after low-intensity BFR only (21). lnLF significantly increased for low-intensity and low-intensity BFR compared to the high-intensity session at 10-min post-exercise. High-intensity and low-intensity BFR lnHF significantly decreased up to the 1-h post-exercise time period. High-intensity measures were significantly lower than low-intensity BFR and low-intensity sessions for up to 30-min post-exercise (21).

It is difficult to directly compare post-exercise autonomic responses across protocols given differences in repetitions, sets, rest periods, amount of weight used across different studies (11). In addition, total exercise volume, BFR cuff pressure, cuff size, and whether the cuff pressure was released during the rest interval (e.g., intermittent) or maintained during the rest interval (e.g., continuous) makes comparison to the literature difficult. Nonetheless, the present investigation used intermittent BFR application. Previous studies have shown that muscle activation in muscle distal to cuff placement is not different between intermittent and continuous BFR protocols (23) and intermittent BFR application may be associated with less swelling or metabolic stress (11), which may be advantageous for clinical populations or those that cannot tolerate high perceptual discomfort. Further, the BFR device used in the present study is considered an autoregulation device (43), in which device engineering allows for precise and rapid control of pressure during different phases of muscle contraction (44). Although research is just beginning to understand these engineering differences (43), non-autoregulated BFR application may enhance hemodynamics and perceptual responses (45) leading to larger disturbances in post-exercise autonomic modulation.

When BFR was applied VL SmO_2_% significantly decreased compared to the control session. Oxygenated hemoglobin was significantly lower when BFR was applied compared to the control session, and inversely deoxygenated hemoglobin was significantly higher during BFR compared to the control session. Shriver et al. (2023) reported similar findings during a walking treadmill protocol in SmO_2_% where LOP (0%, 40%, 80%, & 100%) were randomized during seven stages on both legs at the most proximal location of the leg (46). The VL muscle being monitored during the walking treadmill protocol showed a significant decrease in SmO_2_% at all levels of LOP's. Reis et al. (2019) also investigated how VL tissue SmO_2_% were affected during a unilateral KE exercise (47). Investigators used multiple different LOP (non-BFR, 40%, 60%, and 80%) while occluding venous blood flow. The change in deoxygenated hemoglobin remained similar between 60% and 80% LOP. However, compared to 60% and 80% LOP, non-BFR and 40% LOP resulted in significantly lower deoxygenated hemoglobin during exercise (47). Overall, our study findings are in agreement with the previous studies in that BFR is an effective exercise method to reduce muscle tissue oxygenated hemoglobin and increase deoxygenated hemoglobin during low-intensity exercise. This response suggests greater muscle specific metabolic stress, which in combination with mechanical tension and muscle damage, may be linked with the conditions required to induce muscle hypertrophy (48).

Perceptual intensity of the exercise session was recorded as RPE following the last repetition of each set on both legs. Our study found RPE ratings were significantly higher following BFR-KE than compared to the Control-KE. Shriver et al. (2023) also reported when occlusion pressure was higher it resulted in an increase in RPE (46). Similarly, Bartolomei et al. (2022) also investigated the difference in RPE when comparing high and low occlusion pressures and found that a LOP of 80% had a significantly greater increase in RPE compared to a LOP of 40% during a barbell preacher curl (49). Although there are differences in exercise protocols and intensities between previous studies, it appears that that the greater LOP the larger the increase RPE due to the muscle being in more of a hypoxic state.

Strengths of our investigation included the individualized and objective prescription of BFR cuff pressure at 80% LOP. We also controlled exercise volume between conditions for a robust evaluation of the effect of vascular occlusion on post-exercise autonomic modulation. Further, investigations into intermittent blood flow restriction application are currently limited in the literature. A potential limitation to our reported autonomic modulation findings was that our subjects were asked to breathe normally while lying in a supine position following each exercise condition given the exercise prescription may have influenced metabolic stress and pulmonary ventilation differently. Controlled breathing techniques can help elicit respiratory sinus arrhythmia which activates the parasympathetic nervous system to help facilitate faster recovery (40, 50). The lack of controlled breathing in our study may have limited the ability of sympathovagal balance and could have caused the co-activation between the sympathetic and parasympathetic nervous systems. Additionally, controlled breathing has been shown to affect thoracic stretch receptor afferents, which can cause a reflex inhibition of the autonomic nervous system and influence HRV spectral components (50).

## 6 Conclusion

The findings of our study suggest that single joint KE exercise with intermittent BFR facilitates greater perceptual effort and localized muscular stress; however, there was no apparent influence of vascular occlusion on post-exercise autonomic modulation. Consistent with previous studies, when BFR was applied during exercise it significantly decreases oxygenated hemoglobin and increases deoxygenated in working muscles distal to the cuff inflation. The findings of this study also suggest when BFR was applied it significantly increases perceptual effort compared to the same exercise without BFR. Performing a unilateral KE when BFR is applied could be a useful exercise training method to mitigate longer acute modulations in vagal activity while improving musculoskeletal adaptations.

## Data Availability

The raw data supporting the conclusions of this article will be made available by the authors, without undue reservation.
